# Trends in falls among older adults before and during the COVID-19 pandemic in Ontario, Canada: A retrospective observational study

**DOI:** 10.1186/s12877-024-05032-y

**Published:** 2024-05-11

**Authors:** Ashini Weerasinghe, Justin Thielman, Ye Li, Varsha B. Doguparty, Alexia Medeiros, Sue Keller-Olaman, Sarah Carsley, Sarah A. Richmond

**Affiliations:** 1 Public Health Ontario, 661 University Ave, Suite 1701, Toronto, ON M5G 1M1 Canada; 2 Dalla School of Public Health, University of Toronto, Toronto, Canada

**Keywords:** Time series, Falls, COVID-19 pandemic, Older adults, Hospitalization, ED visits

## Abstract

**Background:**

The public health measures associated with the COVID-19 pandemic may have indirectly impacted other health outcomes, such as falls among older adults. The purpose of this study was to examine trends in fall-related hospitalizations and emergency department visits among older adults before and during the COVID-19 pandemic in Ontario, Canada.

**Methods:**

We obtained fall-related hospitalizations (*N* = 301,945) and emergency department visit (*N* = 1,150,829) data from the Canadian Institute for Health Information databases from 2015 to 2022 for adults ages 65 and older in Ontario. Fall-related injuries were obtained using International Classification of Diseases, 10th edition, Canada codes. An interrupted time series analysis was used to model the change in weekly fall-related hospitalizations and emergency department visits before (January 6, 2015-March 16, 2020) and during (March 17, 2020-December 26, 2022) the pandemic.

**Results:**

After adjusting for seasonality and population changes, an 8% decrease in fall-related hospitalizations [Relative Rate (RR) = 0.92, 95% Confidence Interval (CI): 0.85, 1.00] and a 23% decrease in fall-related emergency department visits (RR = 0.77, 95%CI: 0.59, 1.00) were observed immediately following the onset of the pandemic, followed by increasing trends during the pandemic for both outcomes.

**Conclusions:**

Following an abrupt decrease in hospitalizations and emergency department visits immediately following the onset of the pandemic, fall-related hospitalizations and emergency department visits have been increasing steadily and are approaching pre-pandemic levels. Further research exploring the factors contributing to these trends may inform future policies for public health emergencies that balance limiting the spread of disease among this population while supporting the physical, psychological, and social needs of this vulnerable group.

## Introduction

Falls are common among Canadian older adults (ages 65 and older), with about 20–30% of older adults experiencing a fall in each year [1]. In addition to the immediate and long-term physical impacts including fractures, increased frailty, chronic pain, and the need for injury rehabilitation, falls may serve as a tipping point in the overall decline of psychological and physical health through social isolation, depression, and self-restriction of physical and social activities [2–4].

On March 11, 2020, the World Health Organization declared COVID-19 a pandemic [5] and soon after, the province of Ontario issued a lockdown on March 17, 2020 to limit the spread of SARS-CoV-2 [6]. During this period, only essential services (e.g., grocery stores, pharmacies, and health services) were kept open, while other services (e.g., restaurants, bars, malls, gyms, and in-person learning at schools) were closed [7]. In addition, the government encouraged only those in utmost need to seek care in hospitals, to avoid overwhelming the healthcare system. Early in the pandemic, older adults were identified as a vulnerable group that was at risk of developing serious complications from COVID-19 due to their age and a higher incidence of comorbidities [8]. Older adults were advised to stay-at-home and avoid in-person contact with others. Due to these measures, many older adults were confined to their homes during the lockdown period, where they faced social isolation [9] and reduced opportunities for physical activity [10].

In a USA survey administered in January 2021, 37% of older adults reported a decrease in physical activity since the beginning of the COVID-19 pandemic [10]. Prolonged physical inactivity and sedentary behaviours are associated with decreased muscle mass which impacts strength, mobility and balance [11]. Reduced musculoskeletal strength increases the risk for falls, which can lead to serious injury and result in hospitalizations or emergency department (ED) visits [12].Therefore, while physical activity, especially walking, could increase the risk of falls among older adults, it can also protect against serious injury from falls [13].

Recent studies describing the incidence and prevalence of fall-related hospitalizations and/or ED visits pre-COVID-19 and during COVID-19 had varying results. Eight studies from the US, UK, Israel, Turkey, and Georgia found that there was a reduction in the frequency of falls during the COVID-19 pandemic [14–21]. Five studies from the US, Singapore, Jordan, Turkey and Brazil revealed that admissions due to falls increased during the COVID-19 pandemic in comparison to pre-pandemic years [22–26]. One study from the US found no significant difference in the number of falls among older adults before and during the COVID-19 pandemic [27]. One study from Ireland had mixed results, revealing that while falls decreased in the spring of 2020, they increased with the relaxation of lockdown measures in summer 2020, in comparison to the pre-pandemic period [28]. Most of these studies; however, were limited to a single-center or had small sample sizes. In studies with large sample sizes, authors often did not consider a time series analysis to account for historical trends in the number of falls [15, 18–20]. To address this gap, as well as to examine the impact of the lockdown policies in Ontario, we aimed to examine the trends in hospitalizations and ED visits for falls among older adults in Ontario during the COVID-19 pandemic (2020–2022) compared to the five years that preceded the pandemic (2015–2020).

## Methods

### Data sources

Hospital admission and discharge information in Ontario is collected by the Canadian Institute of Health Information’s hospital abstracting databases (Discharge Abstract Database [DAD], National Ambulatory Care and Reporting System [NACRS]). DAD captures administrative, clinical, and demographic information on all hospital discharges from acute inpatient facilities in Ontario. NACRS contains administrative, clinical, and demographic information for all ED visits and selected outpatient visits in Ontario. Hospitalizations from January 6, 2015 to December 26, 2022 for adults age 65 and older in Ontario, Canada were identified using the DAD (*N* = 304,336). ED visits for the same time period among adults age 65 and older in Ontario were identified using the NACRS (*N* = 1,153,504). In both databases, we extracted all visits that included a falls code (International Classification of Diseases, 10th edition, Canada or ICD-10-CA codes W01-W19) in one of the diagnosis fields, along with the following data elements: age, sex, region of Ontario, primary/most responsible diagnosis, postal code [29]. A full list of codes used to define this outcome is listed in Table 1. Region of Ontario (North, Central East, Eastern, South West, Central West, and Greater Toronto Area) was identified according to the each patient’s postal code [30]. Population estimates based on the census obtained from Statistics Canada [31] (up to 2020) and projections data obtained from the Ontario Ministry of Finance [32] (starting in 2021) were used to impute the monthly population of older adults in Ontario. The projections data used the most recent population estimates released by Statistics Canada as the basis for its projections. Population data for month of year was calculated by interpolating the per cent change in population counts between the calendar year before and after with the annual estimates assigned to July (mid-year).

**Table 1:** List of international classification of diseases, 10th edition, Canada codes that classify fall injuries

W01-W19	External Cause of Injury (Fall)	• **W00**: Fall on same level involving ice and snow • **W01**: Fall on same level from slipping, tripping and stumbling • **W02**: Fall involving ice-skates, skis, roller-skates or skateboards • **W03**: Other fall on same level due to collision with, or pushing by, another person • **W04**: Fall while being carried or supported by other persons • **W05**: Fall involving wheelchair • **W06**: Fall involving bed • **W07**: Fall involving chair • **W08**: Fall involving other furniture • **W09**: Fall involving playground equipment • **W10**: Fall on and from stairs and steps • **W11**: Fall on and from ladder • **W12**: Fall on and from scaffolding • **W13**: Fall from, out of or through building or structure • **W14**: Fall from tree • **W15**: Fall from cliff • **W16**: Diving or jumping into water causing injury other than drowning or submersion • **W17**: Other fall from one level to another • **W18**: Other fall on same level • **W19**: Unspecified fall

### Exposure of interest

The exposure or event of interest is the initial public health response to the COVID-19 pandemic that involved large-scale “lockdowns” and closing of non-essential services. In Ontario, this occurred on March 17, 2020, which is the date we consider the “interruption” in the time series analysis. After March 17, 2020, people were advised to stay home as much as possible and shelter in place to avoid exposure to COVID-19 [6].

### Outcomes

We report the weekly average rate of hospitalizations and ED visits for falls per 100,000 population, pre and during COVID-19 as descriptive statistics. For the pre-COVID period, the number of falls from January 6, 2015 to March 16, 2020 was divided by the 2016 census population and 271 weeks and then multiplied by 100,000. During the COVID-19 period, the number of falls from March 17, 2020 to December 26, 2022 was divided by the 2021 census population and 145 weeks and then multiplied by 100,000. The weekly average fall injury rates were also calculated by sex and by age group.

### Data analysis

The analysis involved three stages. Firstly, duplicate DAD and NACRS IDs with multiple falls per visit were deleted to retain one fall record per visit, so that every record had a unique DAD or NACRS visit (99.2% of all DAD visits and 99.8% of all NACRS visits had a unique visit). Then, records with missing age (*n* = 3 for DAD, *n* = 107 for NACRS) and other/undifferentiated sex (*n* = 6 for DAD, *n* = 40 for NACRS) were excluded. These daily records were summarized into weekly fall counts from 2015 to 2022 for analysis, which resulted in 416 weekly time points (week was used as the unit of analysis).

Secondly, negative binomial regression models were used to estimate the rates of fall-related hospitalizations and ED visits for each age (65–84, 85+) and sex (male, female) group using the entire time period available. Using the estimated rates, the expected count for each week were calculated.

Thirdly, an interrupted time series analysis was used to evaluate the impact of the initial lockdown measures implemented in Ontario (the “interruption”) on fall-related hospitalizations and ED visits and to compare data before the pandemic to during the pandemic. The model estimated the both the change in the level and the change in the slope of hospitalizations and ED visits after the lockdown period comparing to pre-lockdown [33]. The following model was used:

$${\mathrm Y}_{\mathrm i}\sim\mathrm{Poisson}\;\left({\mathrm\mu}_{\mathrm i}{\mathrm E}_{\mathrm i}\right)$$

$$\log\;\left({\mathrm\mu}_{\mathrm i}\right)\;=\;{\mathrm\beta}_0+{\mathrm\beta}_1{\mathrm T}_{\mathrm i}+{\mathrm\beta}_2{\mathrm X}_{\mathrm i}+{\mathrm\beta}_3{\mathrm X}_{\mathrm i}\ast\left({\mathrm T}_{\mathrm i}-\mathrm{TI}\right)+{\mathrm\beta}_4{\mathrm S}_{\mathrm i}+{\mathrm\beta}_5{\mathrm C}_{\mathrm i}$$

where Y_i_ and E_i_ represent the observed and expected count of hospitalization/ED visits for week i, T_i_ = (0,1,2,3,…,415) is the week since beginning of the study (January 6, 2015), X_i_ is a binary indicator for each week (1 indicates lockdown) and TI is the first week of lockdown (March 17, 2020; week 271). X_i_*(T_i_-TI) is an interaction term between the interruption (X_i_) and the time elapsed since the beginning of the interruption (T_i_-TI). The natural log transformation of the age and sex adjusted expected counts E_i_ were included the model as an offset term, seasonality was modeled using S_i_ and C_i_ which are sine and cosine transformation of T_i_ with various frequencies.

To reduce the inflation of type I error due to temporal autocorrelation, a General Estimating Equation (GEE) was used and robust standard errors were estimated with a sandwich estimator [34]. Analyses were performed using PROC GEE, SAS 9.4 (SAS Institute, Cary, NC). Parameter estimates related to time (time and time interaction effects) were scaled up and expressed in yearly terms (multiplied by 52) to better display the magnitude of the effect. This study was approved by the Research Ethics Board at Public Health Ontario (ID 2023 − 004.01) and informed consent was not required.

## Results

Table 2 reports the number and percentage of fall-related hospitalizations and ED visits among Ontarians ages 65 and older, from January 6, 2015 to December 26, 2022. There was a total of 301,945 unique hospitalizations and 1,150,829 unique ED visits in Ontario during this time. More than half of the hospitalizations (56.05% pre-COVID and 56.75% during COVID) and more than two-thirds of the ED visits (67.23% pre-COVID and 67.90% during COVID) were among people ages 65–84. Approximately two-thirds of hospitalizations (64.90% pre-COVID and 63.48% during COVID) and ED visits (63.73% pre-COVID and 63.22% during COVID) were among females. More than one-third of hospitalizations (36.28% pre-COVID and 36.89% during COVID) and ED visits (37.27% pre-COVID and 35.51% during COVID) were from the Greater Toronto Area, the most populous region in Ontario.

**Table 2:** Fall-related hospitalizations and fall-related ED visits among adults 65 + in Ontario from January 2015-December 2022

	DAD (*N* = 301,945)		NACRS (*N* = 1,150,829)	
	Pre-COVID (Jan 6, 2015 - Mar 16, 2020) *N* = 188,738	COVID (Mar 17, 2020 – Dec 26, 2022) *N* = 113,207	Pre-COVID (Jan 6, 2015 - Mar 16, 2020) *N* = 747,433	COVID (Mar 17, 2020 – Dec 26, 2022) *N* = 403,396
**Admit Age**				
65–84	105,780 (56.05)	64,240 (56.75)	502,487 (67.23)	273,924 (67.90)
85+	82,958 (43.95)	48,967 (43.25)	244,946 (32.77)	129,472 (32.10)
**Sex**				
Male	66,246 (35.10)	41,348 (36.52)	271,063 (36.27)	148,386 (36.78)
Female	122,492 (64.90)	71,859 (63.48)	476,370 (63.73)	255,010 (63.22)
**Region of Ontario**				
North	14,715 (7.80)	8,319 (7.35)	57,424 (7.68)	31,261 (7.75)
Central East	17,220 (9.12)	10,787 (9.53)	65,274 (8.73)	36,812 (9.13)
Eastern	27,272 (14.45)	16,363 (14.45)	115,617 (15.47)	64,854 (16.08)
South West	28,365 (15.03)	16,429 (14.51)	106,415 (14.24)	59,795 (14.82)
Central West	32,692 (17.32)	19,542 (17.26)	124,135 (16.61)	67,416 (16.71)
Greater Toronto Area	68,474 (36.28)	41,767 (36.89)	278,568 (37.27)	143,258 (35.51)

### Hospitalizations

During the pre-pandemic period, the overall weekly average rate of hospitalizations was 30.80 per 100,000 population, decreasing to 29.17 per 100,000 after initial lockdown measures were implemented (Table 3). Similar decreases were seen by sex and age group. For men, the weekly average rate of hospitalizations decreased from 24.00 per 100,000 pre-COVID to 23.47 per 100,000 during COVID. For women, the weekly average rate of hospitalizations decreased from 36.37 per 100,000 pre-COVID to 33.91 per 100,000 during COVID. Among people ages 65–84, the weekly average rate of hospitalizations decreased from 19.93 per 100,000 pre-COVID to 18.98 per 100,000 during COVID. For people ages 85+, the weekly average rate of hospitalizations decreased from 101.10 per 100,000 pre-COVID to 98.47 per 100,000 during COVID.

**Table 3:** Weekly average rate of hospitalizations and ED visits for falls per 100,000 population

	Hospitalizations		ED visits	
	Pre-COVID (Jan 6, 2015 - Mar 16, 2020)	During COVID (Mar 17, 2020 - Dec 26, 2022)	Pre-COVID (Jan 6, 2015 - Mar 16, 2020)	During COVID (Mar 17, 2020 - Dec 26, 2022)
Overall	30.80	29.17	121.98	104.08
Male	24.00	23.47	98.21	84.32
Female	36.37	33.91	141.45	120.47
Age 65–84	19.93	18.98	94.68	81.04
Age 85+	101.10	98.47	298.51	260.67

**Fig. 1:**
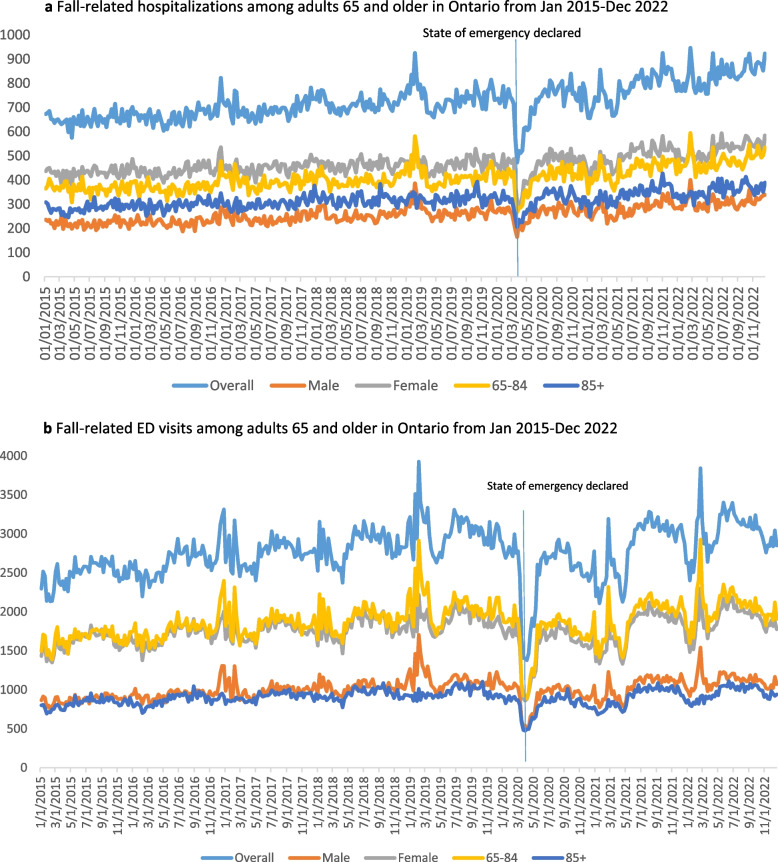
**a** Fall-related hospitalizations among adults 65 and older in Ontario from Jan 2015-Dec 2022. **b** Fall-related ED visits among adults 65 and older in Ontario from Jan 2015-Dec 2022

After controlling for seasonal effects, hospitalization for falls remained relatively stable week-to-week before the pandemic [Relative Rate (RR) = 1.00, 95% Confidence Interval (CI): 1.00, 1.00; Table 4; Fig. 1a). After controlling for the pre-pandemic trend, there was an 8% reduction in fall-related hospitalizations among older adults in Ontario following the onset of the COVID-19 lockdown policy (RR = 0.92, 95% CI: 0.85, 1.00). This was followed by an increasing post-lockdown trend, after the initial decrease associated with the pandemic onset (RR = 1.06, 95% CI: 1.00, 1.13).

**Table 4:** Results of the interrupted time series model for falls-related hospitalizations and ED visits

	Hospitalizations Relative Rate (95% Confidence Interval)	ED visits Relative Rate (95% Confidence Interval)
Time	1.00 (1.00, 1.00)	1.02 (1.00, 1.04)
Interruption (During vs. Pre COVID-19)	0.92 (0.85, 1.00)	0.77 (0.59, 1.00)
Time*Interruption (During vs. Pre COVID-19)	1.06 (1.00, 1.13)	1.08 (1.00, 1.17)

### Emergency department visits

During the pre-pandemic period, the overall weekly average rate of ED visits was 121.98 per 100,000 population, decreasing to 104.08 per 100,000 post-lockdown (Table 3). Similar decreases were seen by sex and age group. For men, the weekly average rate of ED visits decreased from 98.21 per 100,000 pre-COVID to 84.32 per 100,000 during COVID. For women, the weekly average rate of ED visits decreased from 141.45 per 100,000 pre-COVID to 120.47 per 100,000 during COVID. Among people ages 65–84, the weekly average rate of ED visits decreased from 94.68 per 100,000 pre-COVID to 81.04 per 100,000 post-lockdown. For people ages 85+, the weekly average rate of ED visits decreased from 298.51 per 100,000 pre-COVID to 260.67 per 100,000 post-lockdown.

After controlling for seasonal effects, ED visits for falls remained relatively stable week-to-week before the pandemic (RR = 1.02, 95% CI: 1.00, 1.04; Table 4; Fig. 1b). After controlling for the pre-pandemic trend, there was a 23% reduction in fall-related ED visits among older adults in Ontario following the onset of the COVID-19 pandemic (RR = 0.77, 95% CI: 0.59, 1.00). This was followed by an increasing post-interruption trend, after the initial decrease associated with the pandemic onset (RR = 1.08, 95% CI: 1.00, 1.17).

## Discussion

To our knowledge, this is the first paper to examine changes in hospitalizations and ED visits for falls among older adults, pre to during the pandemic, while controlling for trends in fall injury visits over time. We identified marked decreases in both hospitalizations and ED visits immediately following the COVID-19 policy implemented on March 17, 2020. Fall-related hospitalizations dropped by 8% and ED visits dropped by 23%. Following this decrease, both hospitalizations and ED visits gradually increased over time; 0.1% per week for hospitalizations and 0.2% for ED visits. We focused on reporting confidence intervals rather than statistical significance as we are using administrative data.

The sharp drop in fall-related hospital visits during COVID-19 can be explained by a couple of factors. Ontario implemented a number of public health measures to curb the rate of COVID-19 infection and death, including a “lockdown” of non-essential services in March 2020 [6]. People were advised to isolate inside their homes as much as possible. While hospitals and EDs remained open, people were likely hesitant to seek treatment unless absolutely necessary due to fears of COVID-19 transmission. It is conceivable that many older adults avoided seeking care for less severe fall-related injuries which may explain why fall-related ED visits dropped more than hospitalizations. Additionally, while isolating inside their homes, there may have been less opportunity for older adults to participate in the types of activities that can result in an injurious fall. Following the decrease in injury rates shown in March 2020, the gradual increase in both hospitalizations and ED visits for falls may be related to the gradual easing of restrictions and resulting increases in activity, coupled with less fear of contracting COVID-19 due to attending to hospital. Further, due to the reduction of physical activity during the pandemic, the risk of falling may have further increased due to losses of muscle strength, power and balance that can occur during periods of inactivity [35].

Our results add to an emerging body of evidence on the impact of COVID-19 on falls among older adults. Studies published from the early days of the pandemic demonstrate mixed results, with some studies finding a post-COVID-19 increase in falls among older adults [22–26], some finding a decrease [14–21], and others finding no significant difference [27]. Our results are consistent with the majority of previous studies, demonstrating a decrease in falls following the lockdown measures implemented due to COVID-19. The majority of the early studies were restricted to data from one hospital, thus analyzed data from small sample sizes. Further, these studies did not account for trends in fall-related visits that were happening independent of COVID-19.

### Strengths and limitations

The following strengths enhance confidence in our findings. Using data from Ontario’s population based health administrative databases, we were able to identify all fall-related hospitalizations and ED visits in Ontario from 2015 to 2022, amounting to hundreds of thousands of visits, representative of the population. We also conducted a time-series analysis, which allowed us to control for trends in fall-related visits that are independent of the pandemic, as well as seasonal fluctuations in falls and changes in the population of adults over 65. While some published studies using an interrupted time series analysis model use the product between the time since the beginning of the study period and the date of the interruption as the post-interruption slope [33], this can result in a biased estimate of the immediate level change in the time series [36]. Our study used the product between the interruption and the time elapsed since the beginning of the interruption to account for this bias. However, several limitations to our study must be considered. While time-series analysis is not prone to confounding by factors that remain constant over time [33], there are potential time-varying confounders that we did not account for, such the number of previous falls, medication use, clinical characteristics of the study population, or whether they were living in the community or in a long-term care home. Additionally, we adjusted for seasonal fluctuations in falls using the sine and cosine terms in the models, but several peaks in the data remained that we did not account for in the models; however, as these peaks are unrelated to COVID-19 they are unlikely to bias the study’s results. We were not able to adjust for precipitation in the models as well, a known risk factor for falls, due to high levels of missingness in the data available [37]. Finally, the results of this study may not be generalizable to findings outside of Ontario that had different public health measures related to COVID-19.

## Conclusions

Our findings showing a sharp decrease in falls among older adults after the onset of the COVID-19 lockdown policy in Ontario. This was followed by a gradual increase after March of 2020 continuing to the end of the study period. The decrease in hospitalizations and ED visits may be an indication of two important behavioural changes related to the restrictions needed to combat COVID-19: the lockdown policy forced older adults to self-isolate, resulting in a decrease in physical activity, as well as created a delay in seeking treatment due to a fall. These two behaviours have consequences that are not unique to falls. For instance, the social isolation that resulted from staying home has had other negative impacts such as increase in loneliness and sedentary behaviour [38]. As well, delaying treatment likely occurred for injuries or conditions other than those resulting from falls, and prognoses can be worse if treatment is delayed [39]. For instance, Ontario’s cancer screening programs delivered 41% less screening tests in 2020 in comparison to 2019 and cancer screening volumes for most programs remained more than 20% below historical levels by the end of 2020 [40]. Public health restrictions were needed to reduce infection and death due to COVID-19, but this study highlights one of the indirect impacts that should be mitigated during such restrictions.

Other impacts are important to investigate as we may have only seen a decrease in falls due to delays in health care-seeking. Future studies should examine changes in falls before and during the COVID-19 pandemic in comparison to other injury types, as well as to the total number of hospitalizations and ED visits during COVID to examine the type of injuries presenting to hospital. Further, the impact of delayed health care-seeking on health outcomes among older adults should be investigated.

## Data Availability

The data that support the findings of this study are available from the Canadian Institute for Health Information but restrictions apply to the availability of these data, which were used under license for the current study, and so are not publicly available. Data are available from the authors upon reasonable request and with permission of the Canadian Institute for Health Information.
